# Account of a Mineral Spring at Dudley, in Worcestershire

**Published:** 1809-06

**Authors:** W. Weldon

**Affiliations:** No. 10, Wigmore Street


### *Account of a Mineral Spring at Dudley, in Worcestershire.*

ABOUT two miles to the south of the town of Dudley, upon the estate of the Right Honourable Lord Dudley and Ward, is a spring of mineral water that has been famous in the surrounding country from time immemorial, in various scrophulous and cutaneous diseases. It is said to have been used with great success in what are called obstructions of the abdominal viscera, and in various cutaneous diseases.

In a disease that, under one form or other, is so very general throughout Britain, it is probable that this mineral water may prove a valuable and useful remedy, and deserving of a more general attention than hitherto it has met with.

Like most other mineral waters, it would be most advisable to administer it on the spot; but when circumstances render that inconvenient, it may be sent for and used at home.

I am not personally acquainted with the geology of the surrounding country, but I am told it is very interesting. It appears to be of what Werners names the independant coal coal formation. Coals occur in great abundance, one stratum particularly is said to be ten yards in thickness, with iron stone, basaltes, clay, &c.

The saline spring flows into a well, near a ridge of high lands, on the sides of which, at some short distance, coals and iron stone are seen creeping out. The well is about 36 feet in depth, and 7½ feet in diameter. The sides have lately been fenced, to keep out foreign water, which was supposed to run into it, with a dam of bricks set in clay and lined with elm boards. The bottom is a ferruginous argillaceous sand stone, through which is perforated a hole, whence the water issues, and rises to within about four feet from the surface.

The sides of the well, near the top, are covered with a yellowish ochrey substance. When the water is fresh taken up, it is perfectly transparent and colourless. It is little refractive of light, nor can it be said to sparkle; but after standing for a short time numerous small bubbles of air are seen adhering to the bottom and sides of the glass it is taken into. After a time the water becomes rather turbid, and at length a pale ochreous precipitate falls down, leaving the supernatant water transparent.

In large quantity the water smells of sulphurated hydrogen; but if half a pint or less be examined apart, the odour is hardly perceptible.

When the temperature of the atmosphere indicated 40° of Fahrenheit, some water just taken from the well raised the thermometer to 47½°. At another time, when the temperature of the surrounding air was 75°, water taken from near the surface of the well lowered the thermometer to 56°, and a portion taken from the bottom to 52°.

The taste of this water very much resembles the taste of sea water.

The specific gravity of this water is found to differ at different depths from the surface. It differs also very considerably at different times. Specimens have been sent to me at four different periods. The following are the dates and specific gravities:

July 18, 1806, taken from the bottom of the well, sp. g. to distilled water as 1018 to 1000.

Jan. 23, 1808, from the bottom of the well, sp. g. as 1028.8 to 1000.

Oct. 27, 1808, sp. g. as 1013.5 to 1000.

Dec. 2, 1808, two portions of the mineral water were sent to me, one of which was taken from about the middle of the well, the other from the bottom.

Sp. g.

Sp. g. of the water from the bottom 1013.5.

Sp. g. of the water from the middle 1012.

In consequence of some fresh water springs being found to discharge themselves into the well, it has lately been emptied and walled round as above stated, and it is probable that hereafter the strength of the water will be more regular.

It seems that the workmen experienced a great deal of trouble in securing the dam. At first they built the dam with common lime mortar. The action of the water destroyed its cementing power. Then a fresh wall was built with a grey lime, which the neighbourhood affords, and which sets very firm under water, but this also, as might be expected, failed from the action of the water on the lime. At length the dam was formed by setting bricks in a strong clay that is free from lime, or nearly so, and it seems to answer the purpose intended.

The mineral water that was subjected to the following experiments was taken from the bottom of the well, by sinking empty bottles provided with valves. As soon as the bottles were drawn up they were carefully corked and tied down with bladder.

1. A glass bottle full of this water was perfectly transparent and without sediment while uncorked. After standing for some time in the laboratory, where the thermometer stood at 40°, the cork was drawn, a small portion of gas compressed in the neck of the bottle escaped, but no bubbles of air were seen to escape, or even to form for some minutes after. At length, however, the water became turbid, and gradually deposited an ochrey sediment.

2. a. Pure distilled water was tinctured with a certain proportion of tincture of red cabbage, and placed as a standard of the colour.

6. An equal quantity of the tincture of cabbage was added to a corresponding portion of the mineral water, which was quite fresh. At first it became slightly reddened, but the tint gradually went off, and in three hours the colour was distinctly blued.

a. An equal quantity of the mineral water, which had been boiled and filtered, produced no change of colour of the tincture of cabbage at first, but when examined ten minutes afterwards, it was slightly blued.

3. a. Pure distilled water was coloured with a certain proportion of tincture of litmus as a standard.

b. The fresh mineral water in the same proportion produced the slightest tint of red. It soon vanished.

c. The c. The mineral water which had been boiled and filtered, produced no changes.

4. a. Pure distilled water was tinged with infusion of rhubarb, in given proportion, as a standard.

6. The fresh mineral water tinged with an equal proportion was very slightly deepened.

c. The boiled water tinged as above, could not be distinguished from pure distilled water.

5. Corresponding experiments were made with infusion of brasil. Both the fresh and the boiled mineral water produced the slightest tint of blue.

6. Syrup of violet was changed to a green by the fresh water. The boiled water produced no change on it.

7. a. A few drops of infusion of gall added to the fresh mineral water produced a violet colour; the water gradually became opaque, and in twenty-four hours had thrown down a rather copious precipitate of a violet red colour. The liquid above was transparent and colourless.

6. The infusion of gall produced no change in the mineral water after boiling.

8. a. Lime water in very small quantity produced a pale ochrey precipitate in the fresh mineral water.

b. On adding to the fresh mineral water an equal part of lime water, a white precipitate formed, which dissolved again on adding nitrous acid.

9. Strong sulphuric acid rendered the fresh water turbid, and disengaged a number of air bubbles. An abundance of sulphate of lime formed.

10. A few drops of diluted sulphuric acid was added to some of the mineral water, which had been boiled and filtered, and was previously warmed. No flavour of sulphurated hydrogen was perceptible.

11. The mineral water previously boiled was treated, as in 10, with muriatic acid. No change was produced in its transparency, nor was any flavour perceptible except of the muriatic acid.

12. Silver leaf left in contact with a portion of the fresh water, in a closed vial, for several days, was not perceptibly tarnished.

13. Nitrate of quicksilver produced a copious white precipitate.

14. a. Oxyd of bismuth mixed with the fresh water in the proportion of gr. 4 to 4 dr. was very slightly darkened.

b. With the boiled water no change of colour was produced.

15. a. Acetate of lead threw down from the fresh water a dense dense white precipitate. When the precipitate had wholly subsided, the surface of it was of a pale ochre or fawn colour, apparently produced by the oxyde of iron only.

b. With the mineral water after being exposed to the atmosphere for a few hours and decanted, the precipitate was a pure white.

16. Nitrate of lead threw down a dense precipitate of a ure white colour.

17. Nitric acid produced no change in the fresh water.

18. Nitrate of silver produced a dense white precipitate, which was kept for several days in a dark room without undergoing the least change of its colour.

19. a. A few drops of acetite of silver threw down a precipitate of a rather dull white colour. It was kept in a dark room for 24 hours, when it appeared somewhat darkened.

b. The boiled water treated in the same manner, did not darken the acetite of silver.

20. a. Muriate of quicksilver added to the fresh water, produced no immediate change. After a few minutes the water became slightly opaque, and a slight precipitate at length appeared. It scarcely exceeded the quantity of oxide of iron which the water contained; but the colour was of a redder tint than the ochrey powder which the water let fall spontaneously.

b. The same salt added to some of the mineral water which had been exposed to the atmosphere for some days and decanted, produced no change in it.

21. Oxalate of ammonia instantly produced a copious white precipitate.

22. Oxalic acid produced the same.

23. A solution of pure ammonia threw down a precipitate.

24. Equal parts of the mineral water and of fresh made lime water were mixed together. The precipitate that was thrown down was separated and washed. It was dissolved in muriatic acid, precipitated by subcarbonate of soda, washed and dried in a low heat. Distilled vinegar dissolved a part of it. A solution of pure potash dissolved the greater part of the remainder, leaving a brown powder.

25. Acetate of barytes produced no precipitate in the filtered water.

26. Muriate of barytes produced no precipitate in 24 hours.

When the saline contents of the water were concentrated by evaporation to one fourth, a slight precipitate formed.

27. Muriate

27. Muriate of strontian produced no precipitate in the water, either when fresh or when concentrated, as in 26.

28. Phosphate of soda produced a copious precipitate.

29. Carbonate of ammonia produced a white cloud.

30. A crystal of carbonate of potash did the same.

31. In the fresh water, succinate of ammonia produced a reddish brown precipitate.

b. In the boiled water, a few drops of succinate of ammonia produced no change.

32. a. Prussiate of potash and iron produced a deep blue precipitate in the fresh water.

b. In the boiled water, this triple salt produced no change.

33. A portion of the fresh water was boiled in a glass retort, the curved arm of which was placed under an inverted jar filled with quicksilver. The gas which was thus collected, was tried with nitric acid. The acid absorbed a part of it.

34. A portion of gas, produced as in 33, was exposed to the action of a solution of superacetate of lead; by which a part of it was absorbed.

35. Another portion of this gas, and also the residua of the experiments 33 and 34, were severally exposed to the action of lime water. A corresponding proportion from each of the gasses was absorbed.

36. The precipitate obtained by boiling a quantity of the fresh mineral water to about one half, was washed, dried, and treated with diluted muriatic acid. A part of it only was dissolved.

37. A solution of pure ammonia threw down a white precipitate from the muriatic solution obtained in 36.

38. Oxalate of ammonia, occasioned a precipitate in the same solution.

39. The powder which resisted the action of the muriatic acid in 36, was boiled in subcarbonate of soda, and then in a solution of pure potash. A part of it was dissolved by each.

40 The powder left undissolved by the last operation, was boiled in muriatic acid. What remained was melted in a large proportion of pure potash. Silex was precipitated on the addition of distilled water.

41. A quantity of the fresh water was evaporated to dryness. The dry mass was treated with successive portions of alcohol, the specific gravity of which was 815°. The residuum, which resisted the action of the alcohol, was dissolved solved in water, filtered, and slowly evaporated. The crystaline substance then successively separated was pure common salt.

From these experiments, it appears that this mineral water contains a small proportion of sulphurated hydrogen, the same of free or rather super-combined carbonic acid, carbonate of iron, carbonates of lime, of magnesia, and of argil, probably carbonate of manganese also; a minute proportion of sulphate of lime, with muriates of soda, lime, magnesia, and of argil, silica with azot or atmospheric air.

To obtain an approximation to their respective proportions, the following additional experiments were made.

42. Grains by weight 22045 = to 85.973 cubic inches nearly of the fresh mineral water were poured into a flask, and securely luted to a tube, terminating under a graduated jar filled with quicksilver.

The quantity of atmospheric air that was contained above the water in the flask, and in the tube, was ascertained to be 9.5 cubic inches. The thermometer was at 47° of Fahrenheit, the barometer 29.7. The water was boiled for about half an hour. When the apparatus was cold, the whole of the air in the flask and tube was transfered into the graduated jar. When reduced to the same temperature and barometrical pressure as before the operation of boiling, it measured 16.1 cubic inches.

43. The gas thus collected was washed in a solution of super-acetate of lead, until no further absorption took place. The quantity absorbed was 1.95 cubic inches.

44. The gas was then exposed to the action of fresh made lime water, until it underwent no further diminution of bulk. The quantity absorbed by the lime water was 2.1 cubic inches.

45. The residuum gas, amounting to 12.05, was next treated with a solution of sulphurate of lime, that had been saturated with azot. The quantity of oxygen absorbed was 1.9 cubic inches.

The 9.5 cubic inches of atmospheric air contained in the apparatus consisted of 7.5 cubic inches of azot, and 2 cubic inches nearly of oxygen. The whole of the oxygen indicated in 45 was contained therefore in the atmospheric air. The small deficiency of oxygen appears to have been produced by that quantity having combined with the black oxide of iron, which during the experiment, fell down in form of red oxide.

2.65 cubic inches of azot therefore was given out by the quantity before stated of the mineral water.

46. 88180

46. 88180 grains by weight = to 343.9 cubic inches of the fresh mineral water, were boiled for an hour or more, and filtered. They were then evaporated to about 30 cubic inches, when the muriate of soda began to crystalise. The water was then filtered again; and the small quantity of precipitate that was then obtained, and which from a previous examination was known not to contain any sulphate of lime, was added to the former.

47. The precipitates obtained from the mineral water by 46, were treated with strong muriatic acid, and boiled in it, which did not completely dissolve them. The undissolved powder was washed and boiled for some time in a silver crucible, with a strong solution of pure potash. The solution was diluted, the powder separated, and again treated with muriatic acid, washed and dried in a red heat. This powder weighed grains 2.6.

48. The washings were all added to the solution formed in 47, and the whole precipitated by sub-carbonate of soda. The supernatant solution was neutralised, and the precipitate washed. It was re-dissolved in muriatic acid. The muriatic solution was neutralised, diluted, and treated with prussiat of potash and iron. The precipitate thus formed, was calcined to destroy the prussic acid; nitric acid was abstracted from it two or three times, and then it was dissolved in muriatic acid; the solution was high coloured; it was diluted with water, and precipitated by carbonate of potash. The precipitate was separated by filtration, washed and dried. The subcarbonate of iron thus obtained weighed gr. 5.5.

49. The liquor left after filtering off the subcarbonate of iron in the last operation, was boiled for three quarters of an hour or more; a light coloured precipitate after some time began to separate, which soon became darker coloured. This powder, which I conclude to be carbonate of manganese, when washed and dried, weighed gr. 0.75.

50. The muriatic solution that was filtered off from the prussiate in 48, was accurately neutralized. Oxalate of ammonia was then dropped in gradually, as long as a precipitate formed. The oxalate of lime when washed and dried, weighed gr. 3.1 = g. 1.5 of pure lime, or gr. 3.1 of carbonate of lime.

51. The solution left in 50, was then treated with pure potash. The precipitate was separated, washed, and redissolved in muriatic acid, and again precipitated by subcarbonate of soda, washed and dried in a heat of near 200. It weighed gr. 19.75.

52. This

52. This precipitate was treated with distilled vinegar, until the vinegar no further acted upon it. The residuum when washed and dried, weighed gr. 3.2. Consequently, gr. 16.55 of carbonate of magnesia was dissolved by the vinegar, and gr. 3.2 of subcarbonate of alumine was left.

53. The concentrated mineral water, which was separated from the earthy carbonate, &c. in 46, was diluted with distilled water, and oxalate of ammonia added until the whole of the lime fell down. The oxalate of lime that was then obtained was well washed and dried. It weighed grs. 482.25=to grs. 241. of pure lime,=to grs. 482.25 of dry muriate of lime, taking Kirwan's estimate for the composition of the latter.

54. Pure potash was now added to the filtered solution left in 53. The precipitate was separated, re-dissolved in muriatic acid, and precipitated by sub-carbonate of soda. The precipitate washed, and dried, weighed grs. 118.3.

55. This precipitate was treated with distilled vinegar, until no more dissolved. The sub-carbonate of magnesia then dissolved weighed grs. 99.05 equal to grs. 143.45 of muriate of magnesia, according to Kirwan. The alumine when heated red hot, washed, and dried, weighed grs. 17.25=to nearly grs. 57.3 of muriate of alumine.

56. 88180 grains of the fresh water were evaporated to dryness. A quantity of muriatic gas escaped towards the end of the operation from the partial decomposition of the muriates of magnesia and alumine. Water and a small quantity of muriatic acid were now added to dissolve the soluble part of the mass. The insoluble powder was then treated with strong muriatic acid, washed, dried, and weighed. It was now thrown into a hot solution of sub-carbonate of soda, from which it was separated and treated with diluted muriatic acid, which dissolved a minute proportion of carbonate of lime. This was precipitated and separated by a filter, but did not exceed 0.25 of a grain, by computation.

57. The insoluble powder which could consist only of silica and alumine, was boiled in a silver crucible with a solution of pure potash. The solution was decanted off, and the white powder that was left was washed and exposed to a red heat. It weighed grs. 3.

58. In order to obtain a nearer approximation to the quantity of sulphate of lime contained in a given portion of the mineral water, than that which was obtained in 56, when in dissolving the muriates, a part of the sulphate was was probably re-dissolved, 22045 grains of the mineral water were evaporated to about one-eighth of its bulk filtered. A known proportion of muriate of barytes was then added. A slight cloudiness was perceived in a few minutes. Twenty-four hours after, the small quantity of precipitate that had formed was separated by a filter, washed and dried. Allowing for what was lost upon the filter, it amounted to 0.25 nearly.

59. 22045 of the mineral water was exposed for some days to the atmosphere and filtered, nitrate of silver was then added until no further precipitation ensued. The whole was poured upon a filter, and the precipitate was well washed and dried for twenty-four hours in a stove at 212 nearly. The muriate of silver thus formed, weighed 937.25 grs. which, multiplied by 4, gives grains 3749 for the quantity of muriate of silver formed by the muriatic acid contained in 88180 of the mineral water.

60. If we suppose the muriate of silver to contain 18 per cent. of acid, the quantity of muriatic acid would amount to 674.82 grains.

| | |
|---|---|
| If 482.25 of muriate of lime, contain | grs. 202.545 |
| 143.45 of muriate of magnesia | 81.766 |
| 57. 3 of muriate of alumina | 17. 19 |

of muriatic acid; there will be grs. 787 of muriate of soda, supposing it to contain 50 per cent. of base, 44 of acid, and 6 of water.

61. A given quantity of the mineral water was evaporated to dryness, and digested in alcohol, 815° applied in successive portions to dissolve the earthy muriate. The muriate of soda then obtained, was decomposed by nitrate of silver. The result of this experiment was nearly as above. The following, therefore, may be considered as a near approximation to the quantity of gaseous and solid contents contained in 88180 grains, or 343.9 cubic inches of the mineral water.

| | Cubic inches. |
|---|---|
| Of sulphurated hydrogen | 7. 8 |
| carbonic acid | 8. 4 |
| azot | 10. 6 |
| Of muriate of lime, grains | 482.25 |
| of magnesia | 143.45 |
| of alumina | 573. |
| of soda | 787. |
| Of sulphate of lime | 1.07 |
| carbonate of lime | 3. 1 |
| of magnesia | 16.55 |

| | |
|---|---|
| Subcarbonate of alumina - - - - - - - - - - | 3. 2 |

Subcarbonate of red oxide of iron, grs. 5.5 is nearly equal to grs. 5. of the subcarbonate of black oxide.

| | |
|---|---|
| Of subcarbonate of manganese - - - - - - | 0.75 |
| Of silica - - - - - - - - - - - - - - - - | 3. |

Hence one gallon, or 231 cubic inches of the water contained,

| | |
|---|---|
| Of sulphurated hydrogen, cubic inches - - | 5.24 |
| carbonic acid - - - - - - - - - - - - | 5.64 |
| azot - - - - - - - - - - - - - - - - | 7. 1 |
| muriate of lime - - - - - - - - - - - - | 323.93 |
| of magnesia - - - - - - - - - - | 96.35 |
| of alumina - - - - - - - - - - | 38. 5 |
| of soda - - - - - - - - - - - - | 528.63 |
| Of sulphate of lime - - - - - - - - - - | 0.71 |
| carbonate of lime - - - - - - - - - - | 2. 1 |
| of magnesia - - - - - - - - | 11.12 |
| Of subcarbonate of alumina - - - - - - - | 2.15 |
| of black oxide of iron - - | 3.36 |
| of manganese - - - - - - | 0.475 |
| Of silica - - - - - - - - - - - - - - - | 2. |

I have before observed, that this mineral water has been found to possess different specific gravities at different times, consequently to contain at different times, different quantities of ingredients. The specimen of the water that was sent to me in July, 1806, the specific gravity of which was 1018, was submitted to some experiments, which shew that it differs also at different times in the proportion of its ingredients. In this case the difference is very remarkable in relation to the oxide of iron.

When boiled in a retort that terminated under quicksilver, the black oxide of iron fell down in abundance, and the earthy carbonates were in very small proportion. The water that was decanted, threw down a green hydrate of iron mixed with magnesia, on the addition of lime water. When the water was boiled in the open air and filtered, lime water threw down a brown precipitate.

The following is the proportion of some of the ingredients it contained. While this examination was proceeding, I was obliged to suspend it for a time to attend to more pressing avocations, and an accident prevented my completing it.

From a wine gallon, or 231 cubic inches, was obtained,

| | |
|---|---|
| Of muriate of soda - - - - - - - | grs. 843. |
| of lime - - - - - - - | 311. |
| magnesia and alumina - - | 145. |

Of

| | |
|---|---|
| Of muriate of iron - - - - - - - - - - | 26. |
| Of carbonate of iron - - - - - - - - - - | 9. |
| silica - - - - - - - - - - - - - - - - | 0.75 |
| Of earthy carbonates, about - - - - - - - | 4.5 |
| Of carbonic acid and sulphurated hydrogen, (the latter in small proportion,) cubic inches - - - - - | 23.735 |
| Of azot - - - - - - - - - - - - - - - - - | 12. |

In both of the foregoing statements, it will be seen that I have given the ingredients, as they occurred on decomposition. But it by no means follows that they exist in that state when in solution. It is more probable, that when in solution the earths and oxides are combined with the acids, as is observed by Mons. Berthollet, in proportion, depending upon a power produced by the respective proportions and the respective affinities of all the ingredients towards each other.

W. WELDON.

*No.* 10, *Wigmore Street, March* 8, 1809.

